# Supplementary material for: The molecular basis of sugar detection by an insect taste receptor
Source: Nature. 2024 Mar 6;629(8010):228–34. doi: 10.1038/s41586-024-07255-w (PMC11062906; doi:10.1038/s41586-024-07255-w)
Supplement: Supplementary file 1 — Raw gel images for SDS–PAGE and native-PAGE gels presented in Extended Data Figs. 5c,d and 7c,d. [file 41586_2024_7255_MOESM1_ESM.pdf]

nature portfolio

https://doi.org/10.1038/s41586-024-07255-w

Supplementary information

The molecular basis of sugar detection by an insect taste receptor

In the format provided by the authors and unedited

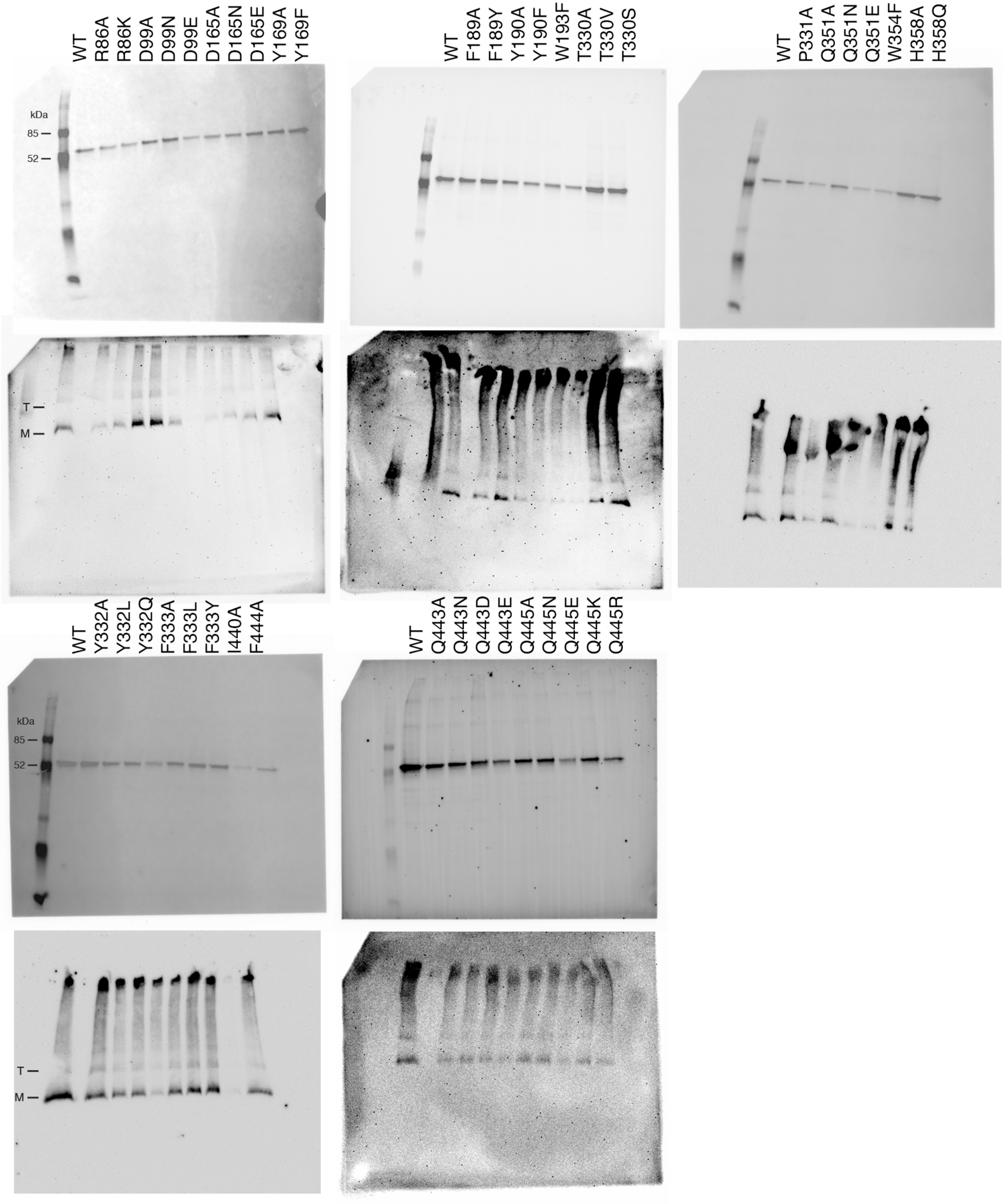
WT R86A R86K D99A D99N D99E D165A D165N D165E Y169A Y169F
kDa
85
52
T
M
WT F189A F189Y Y190A Y190F W193F T330A T330V T330S
WT P331A Q351A Q351N Q351E W354F H358A H358Q
WT Y332A Y332L Y332Q F333A F333L F333Y I440A F444A
kDa
85
52
T
M
WT Q443A Q443N Q443D Q443E Q445A Q445N Q445E Q445K Q445R
