# Supplementary material for: The molecular basis of sugar detection by an insect taste receptor
Source: Nature. 2024 Mar 6;629(8010):228–34. doi: 10.1038/s41586-024-07255-w (PMC11062906; doi:10.1038/s41586-024-07255-w)
Supplement: Supplementary file 2 — Reporting Summary [file 41586_2024_7255_MOESM2_ESM.pdf]

nature portfolio

Corresponding author(s): Butterwick, J.A.

Last updated by author(s): Feb 17, 2024

Reporting Summary

Nature Portfolio wishes to improve the reproducibility of the work that we publish. This form provides structure for consistency and transparency in reporting. For further information on Nature Portfolio policies, see our Editorial Policies and the Editorial Policy Checklist.

Statistics

For all statistical analyses, confirm that the following items are present in the figure legend, table legend, main text, or Methods section.

n/a	Confirmed	
☐	☒	The exact sample size (*n*) for each experimental group/condition, given as a discrete number and unit of measurement
☐	☒	A statement on whether measurements were taken from distinct samples or whether the same sample was measured repeatedly
☐	☒	The statistical test(s) used AND whether they are one- or two-sided<br>*Only common tests should be described solely by name; describe more complex techniques in the Methods section.*
☒	☐	A description of all covariates tested
☐	☒	A description of any assumptions or corrections, such as tests of normality and adjustment for multiple comparisons
☐	☒	A full description of the statistical parameters including central tendency (e.g. means) or other basic estimates (e.g. regression coefficient) AND variation (e.g. standard deviation) or associated estimates of uncertainty (e.g. confidence intervals)
☐	☒	For null hypothesis testing, the test statistic (e.g. *F*, *t*, *r*) with confidence intervals, effect sizes, degrees of freedom and *P* value noted<br>*Give P values as exact values whenever suitable.*
☒	☐	For Bayesian analysis, information on the choice of priors and Markov chain Monte Carlo settings
☒	☐	For hierarchical and complex designs, identification of the appropriate level for tests and full reporting of outcomes
☒	☐	Estimates of effect sizes (e.g. Cohen's *d*, Pearson's *r*), indicating how they were calculated

Our web collection on statistics for biologists contains articles on many of the points above.

Software and code

Policy information about availability of computer code

Data collection	SerialEM 4.1-beta, PTI Felix 1.42b, FLIPR Tetra
Data analysis	Relion 3.1, cryoSPARC 3.3.1, CTFFIND 4.1, MotionCor2, ChimeraX 1.7, PHENIX 1.20.1-4487, HOLE, Coot 0.9.8.1, PyMOL 2.5.0, GraphPad Prism 10.0.2, JalView 2.11.3.2, ChemDraw 22.2.0, Google Colab, AlphaFold2

For manuscripts utilizing custom algorithms or software that are central to the research but not yet described in published literature, software must be made available to editors and reviewers. We strongly encourage code deposition in a community repository (e.g. GitHub). See the Nature Portfolio guidelines for submitting code & software for further information.

Data

Policy information about availability of data

All manuscripts must include a data availability statement. This statement should provide the following information, where applicable:

- Accession codes, unique identifiers, or web links for publicly available datasets
- A description of any restrictions on data availability
- For clinical datasets or third party data, please ensure that the statement adheres to our policy

The final cryo-EM maps have been deposited in the Electron Microscopy Data Bank under accession numbers EMD-42629 (bound to D-fructose), EMD-42628 (unbound), and EMD-43548 (bound to L-sorbose). The final models have been deposited in the Protein Data Bank under accession numbers 8UVU (bound to β-D-fructopyranose and β-D-fructofuranose, at 75% and 25% occupancy, respectively), 8UVT (unbound), , and 8VV3 (bound to [illegible]-L-sorbopyranose). Coordinates for Orco

and MhOr5, used for structural comparisons in this paper, are deposited under accession numbers 6C70 (Orco), 7LIC (MhOr5), and 7LID (MhOr5 bound to eugenol). Source activity and binding data are provided with this paper. For all other data requests, please contact J.A.B.

Research involving human participants, their data, or biological material

Policy information about studies with human participants or human data. See also policy information about sex, gender (identity/presentation), and sexual orientation and race, ethnicity and racism.

Reporting on sex and gender	Not applicable.
Reporting on race, ethnicity, or other socially relevant groupings	Not applicable.
Population characteristics	Not applicable.
Recruitment	Not applicable.
Ethics oversight	Not applicable.

Note that full information on the approval of the study protocol must also be provided in the manuscript.

Field-specific reporting

Please select the one below that is the best fit for your research. If you are not sure, read the appropriate sections before making your selection.

☒ Life sciences ☐ Behavioural & social sciences ☐ Ecological, evolutionary & environmental sciences

For a reference copy of the document with all sections, see nature.com/documents/nr-reporting-summary-flat.pdf

Life sciences study design

All studies must disclose on these points even when the disclosure is negative.

Sample size	No calculations were performed to determine sample sizes; however, the addition of more data did not alter conclusions from this study.
Data exclusions	No activity or binding data were excluded. Some cryo-EM images and particles were excluded from the final data set following established protocols, as described in the Methods.
Replication	GCaMP activity experiments were repeated on different days (> 4), using independently transfected cells and independently prepared sugar/sweetener solutions. Trp fluorescence experiments were repeated on different days, using independently purified samples and independently prepared sugar solutions. Activity and binding data were successfully replicated each time. Structure determinations were carried out using a single data set for each sample (each independently collected over 2 days). Each cryo-EM dataser was split into two subsets and independently used to refine and validate the final model, following established protocols, as described in the Methods.
Randomization	This study did not allocate experimental groups; thus, no randomization was necessary.
Blinding	No blinding was used; all activity and binding data were analyzes using the same methods and all data were included in the results.

Reporting for specific materials, systems and methods

We require information from authors about some types of materials, experimental systems and methods used in many studies. Here, indicate whether each material, system or method listed is relevant to your study. If you are not sure if a list item applies to your research, read the appropriate section before selecting a response.

Materials & experimental systems

n/a	Involved in the study	
☐	☒	Antibodies
☐	☒	Eukaryotic cell lines
☒	☐	Palaeontology and archaeology
☒	☐	Animals and other organisms
☒	☐	Clinical data
☒	☐	Dual use research of concern
☒	☐	Plants

Methods

n/a	Involved in the study	
☒	☐	ChIP-seq
☒	☐	Flow cytometry
☒	☐	MRI-based neuroimaging

Antibodies

Antibodies used	anti-GFP AlexaFluor-488 (ThermoFisher A21311), Goat anti-rabbit IgG-HRP (ThermoFisher A11357)
Validation	All antibodies were from commercial sources; none were independently validated.

Eukaryotic cell lines

Policy information about cell lines and Sex and Gender in Research

Cell line source(s)	Sf9 (ATCC CRL-1711). HEK293S GnTi– (ATCC CRL-3022), HEK293 (ATCC CRL-1573)
Authentication	Cell lines were obtained from commercial sources; none were independently authenticated.
Mycoplasma contamination	Cell lines were not tested for mycoplasma contamination.
Commonly misidentified lines (See ICLAC register)	Not applicable.

Plants

Seed stocks	Not applicable.
Novel plant genotypes	Not applicable.
Authentication	Not applicable.
